# The Emergent Landscape of Detecting EGFR Mutations Using Circulating Tumor DNA in Lung Cancer

**DOI:** 10.1155/2015/340732

**Published:** 2015-09-13

**Authors:** Wei-Lun Huang, Fang Wei, David T. Wong, Chien-Chung Lin, Wu-Chou Su

**Affiliations:** ^1^Department of Internal Medicine, National Cheng Kung University Hospital, College of Medicine, National Cheng Kung University, No. 138, Shengli Road, North District, Tainan City 704, Taiwan; ^2^Institute of Oral Medicine, College of Medicine, National Cheng Kung University, No. 1, University Road, East District, Tainan City 704, Taiwan; ^3^UCLA School of Dentistry, 10833 Le Conte, CHS-Box 951668, Los Angeles, CA 90095-1668, USA; ^4^Institute of Clinical Medicine, National Cheng Kung University Hospital, College of Medicine, National Cheng Kung University, No. 35, Xiaodong Road, North District, Tainan City 704, Taiwan

## Abstract

The advances in targeted therapies for lung cancer are based on the evaluation of specific gene mutations especially the epidermal growth factor receptor (EGFR). The assays largely depend on the acquisition of tumor tissue via biopsy before the initiation of therapy or after the onset of acquired resistance. However, the limitations of tissue biopsy including tumor heterogeneity and insufficient tissues for molecular testing are impotent clinical obstacles for mutation analysis and lung cancer treatment. Due to the invasive procedure of tissue biopsy and the progressive development of drug-resistant EGFR mutations, the effective initial detection and continuous monitoring of EGFR mutations are still unmet requirements. Circulating tumor DNA (ctDNA) detection is a promising biomarker for noninvasive assessment of cancer burden. Recent advancement of sensitive techniques in detecting EGFR mutations using ctDNA enables a broad range of clinical applications, including early detection of disease, prediction of treatment responses, and disease progression. This review not only introduces the biology and clinical implementations of ctDNA but also includes the updating information of recent advancement of techniques for detecting EGFR mutation using ctDNA in lung cancer.

## 1. Introduction

Lung cancer is the leading cause of cancer death since most patients are diagnosed at advanced stage [1, 2]. The identification of oncogenic driver mutations in lung cancer has led to the rapid rise of genotype-directed target therapy such as EGFR tyrosine kinase inhibitors (TKIs) and has shown dramatic clinical benefits [3]. EGFR mutation analysis is performed on tumor cells in biopsy or cytology specimens obtained from bronchoscopy, computed tomography- (CT-) guided biopsy, surgical resection, or drainage from malignant pleural effusions. Sampling tumor tissue other than surgical resection has inevitable limitations. Tumor heterogeneity in single snapshot in time may lead to selection bias. And it may be difficult to obtain enough DNA for EGFR mutation test if biopsy tissue lacks tumor cells [4]. Since initial detection and continuous monitoring of EGFR mutations are needed, the less invasive procedures are still unmet requirements. Blood-borne biomarkers such as circulating tumor cells (CTCs) and circulating tumor DNA (ctDNA) are promising for the detection of somatic mutations derived from malignant tumors [5], since they harbor the same genetic lesions as the primary tumor. Limitation exists on the uncertainty of collection and diversity of phenotypes from CTCs in blood [6]. ctDNA genotyping has the potential to be more widely used than many CTC capture technologies in development for specific purposes because of important advantages of ctDNA genotyping over CTCs for specimen processing. Firstly, CTCs must be separated from the much more abundant hematologic cells in the blood requiring significant laboratory infrastructure to obtain a viable population of CTCs for study. CTCs in circulation encounter substantial apoptosis and fragility leading to variability between different CTC assays. In contrast, most of the ctDNA genotyping methods require a minimum of special handling and do not depend on special equipment. Furthermore, ctDNA could be analyzed together with plasma DNA from normal cells, which is always present in the circulation. Current technologies are sensitive enough to detect tumor-specific somatic mutations, even if the ctDNA fragments represent only a minority of all DNA fragments in the circulation. In this review, we not only introduce the biology and clinical implementations of ctDNA but also include the updating information of recent advancement of techniques for detecting EGFR mutation using ctDNA in lung cancer.

## 2. Source and Biology of ctDNA

### 2.1. Apoptotic and Necrotic Cells

**Figure 1:**
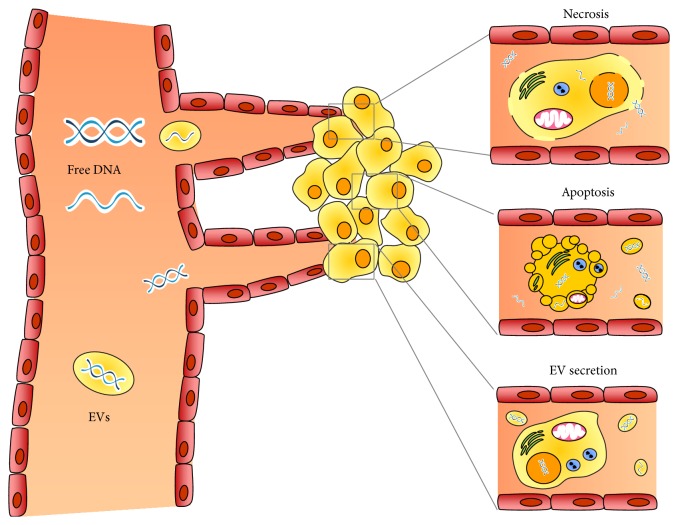
Source and biology of ctDNA.

The finding of circulating extracellular DNA in the bloodstream was first reported at 1948 [7] and the correlation between cell-free nucleic acid levels in plasma and cancer was initially researched in 1977 [8]. It was the first study demonstrating that the plasma levels of circulating free DNA (cfDNA) were much higher in cancer patients than in healthy controls. Tumor cells release small fragments of cfDNA into circulation by multiple mechanisms (Figure 1). The apoptosis and necrosis of cancer cells in the tumor microenvironment are the main explanations for the release of the nucleic acids into the blood [3]. The cellular turnover leads to the increase of apoptotic and necrotic cells as the tumor increases in volume. The apoptotic and necrotic cells are engulfed by macrophages and the digested DNA was released into circulation [9, 10]. When double-stranded ctDNA in plasma is separated and visualized by gel electrophoresis, the fragments with a 180 to 1000 bp size ladder are likely to be formed by apoptosis. In contrast, DNA released by necrosis is nonspecifically digested and thus exhibits smears on electrophoretic separation with fragment sizes about 10,000 bp [10].

### 2.2. Secretion of Extracellular Vesicles

Cells release different types of membrane vesicles of endosomal and plasma membrane origin called exosomes and microvesicles, respectively, into the extracellular environment called extracellular vesicles (EVs) [11]. EVs play an important role of intercellular communication by serving as vehicles for transferring cytosolic proteins, lipids, and nucleic acids between cells. Thus, DNA secreted by EVs has also been suggested as a potential source of ctDNA. Recent investigations provide further evidence that EVs carry not only proteins, mRNA, microRNA, mitochondrial DNA [12], and single-stranded DNA, but also large fragments (>10 kb) of double-stranded carrying mutated KRAS, p53 and EGFR sequences [13, 14]. There are many attractive advantages of EV DNA as a marker. First, EVs are very stable under different conditions that they can protect the DNA cargo against degradation and denaturation in the extracellular environment including the circulation [15]. Second, EVs can be collected from complex plasma samples via various isolating methods such as ultracentrifugation and immunoaffinity isolation based on specific EV surface markers. Thirdly, EVs can be transported via circulation and are found in all kinds of cancer associated body fluids such as pleural effusion, ascites, saliva, and urine [16]. They provide other sources for ctDNA detection other than serum.

## 3. Assays for EGFR Mutations Using ctDNA in Plasma Samples

Since ctDNA often represents a small fraction (<1.0%) of total cfDNA, its detection remains challenging [4]. Thus, direct sequencing approaches like Sanger sequencing or pyrosequencing are not suitable for detecting EGFR mutations using ctDNA. Several different types of PCR-based assays have been explored for ctDNA genotyping including amplification-refractory mutation system (ARMS)/Scorpion assay, digital PCR, mutant-enriched PCR, peptide nucleic acid- (PNA-) mediated PCR, PNA-locked nucleic acid (LNA) PCR clamp, and BEAMing (beads, emulsions, amplification, and magnetics). In addition to the PCR-based assays, mass spectrometry genotyping, high-resolution melting (HRM) analysis, denaturing high performance liquid chromatography (DHPLC), next-generation sequencing (NGS), and electric field-induced release and measurement (EFIRM) were also extensively developed for detecting EGFR mutations in various ctDNA containing cancer associated biofluids including plasma, malignant pleural effusion, and saliva. Here, we review the technical characteristics of these existing technologies shortly and compare their sensitivity, specificity, and predictive value (Table 1).

### 3.1. ARMS/Scorpion Assay

ARMS, also known as allele-specific polymerase chain reaction (ASPCR), is a reliable method for detecting single base mutations or small deletions which is based on the use of sequence-specific PCR primers [17]. This allows amplification of only DNA containing target allele and will not amplify the nontarget allele. Because Taq DNA polymerase is effective at distinguishing between a match and a mismatch at the 3′ end of a primer, specific mutated sequences are selectively amplified. The amplification proceeds with full efficiency, when the primer is fully matched. In contrast, only low-level background amplification occurs when the 3′ base is mismatched. Scorpions are tailed primers containing a PCR primer covalently linked to a probe. The fluorophore in this probe interacts with a quencher which also incorporated in the probe and reduces fluorescence. The fluorophore and quencher become separated when the probe binds to the amplicon during PCR that leads to an increase in fluorescence from the reaction tube [18]. Specific Scorpion ARMS primers have been designed and optimized for detecting various EGFR mutations and have been widely used for ctDNA based assays [19–25].

### 3.2. Digital PCR

Digital PCR is a refinement of conventional PCR that can be used to directly quantify and clonally amplify nucleic acids [26, 27]. It is to amplify a single DNA template from minimally diluted samples and generate amplicons that are exclusively derived from one template. It can be detected with different fluorophores or sequencing to distinguish different alleles. Thus, digital PCR transforms the exponential, analog nature of the conventional PCR into a linear, digital signal, suitable for statistical analysis. Digital PCR has been applied in quantification of EGFR mutants in clinical specimens, providing a promising molecular diagnostic tool [28].

### 3.3. Mutant-Enriched PCR

Mutant-enriched PCR is a sensitive assay that can detect one mutant gene among as many as 10^3^ to 10^4^ copies of the wild-type gene. The sensitivity is achieved by selective PCR amplification of mutant gene sequences with a two stage procedure. The first stage entails the amplification of both mutant and wild-type sequences, followed by selective digestion of only wild-type sequences with thermostable restriction enzymes during PCR. A subsequent step then amplifies the undigested fragments, enriched in mutant sequences [29]. This method has been shown to detect EGFR mutations in various kinds of clinical samples including pleural fluid and surgically resected tissues from patients with NSCLC [30–33].

### 3.4. PNA-Mediated PCR and PNA-LNA PCR Clamp

The assay uses PNA as both PCR clamp and sensor probe. It is a synthetic DNA analog in which the phosphodiester backbone is replaced by a peptide-like repeat [34, 35]. Since PNA contains no charged phosphate groups, the binding between PNA and DNA is stronger than that between DNA and DNA. Since PNA/DNA duplexes are more stable than the relevant DNA-DNA duplexes, PNA will not bind to a not perfectly matched DNA strand. In addition, PNA oligomers are not recognized by DNA polymerases and will not be utilized as primers in subsequence real-time PCR. Thus, the PNA probe binds tightly to perfectly matched wild-type DNA templates but not to mismatched mutant DNA templates and specifically inhibits the PCR amplification of wild-type alleles without interfering with the amplification of mutant DNA. A fluorescein tag also allows the PNA probe to generate unambiguous melting curves for real-time fluorescent monitoring [36]. Oligonucleotides containing LNA hybridize to complementary DNA with an increased affinity compared to oligonucleotide DNA. Thus, the incorporation of LNA residues increases the melting temperature of the oligonucleotide and allows the use of shorter LNA probes as allele-specific tools in genotyping [37]. In PNA clamp PCR, amplification of the wild-type sequences is suppressed and only amplification of the mutant sequences is enhanced. In combination, LNA probes specifically detect mutant sequences in the presence of wild-type sequences. Because PNA clamp primers have wild-type sequences and LNA probes have mutant sequences, they are located in the same position. PNA clamp primers competitively inhibit mutant LNA probes to bind to the wild type, further increasing the specificity of detection. In this way, EGFR mutations can be detected in the presence of 100- to 1,000-fold wild-type EGFR background [38, 39]. Because of its high sensitivity and specificity, PNA-LNA PCR clamp was considered suitable to detect EGFR mutations in histological samples such as surgical specimens as well as in cytological samples such as sputum and pleural effusions [40–43].

### 3.5. BEAMing

BEAMing is a process built on the basis of four of its principal components-beads, emulsion, amplification, and magnetics. BEAMing relies on single-molecule PCR at a massively parallel scale that millions of individual DNA molecules can be assessed in this fashion with standard laboratory equipment, similar to next-generation DNA sequencing technologies [44, 45]. Briefly, BEAMing starts with conventional PCR of a predetermined locus and the PCR product is added to millions of oligonucleotide-coupled beads in oil. An emulsion is then created that most of the beads bind only a single DNA molecule followed by the second round PCR. After the deemulsification and magnetic capture step, single-base primer extension or hybridization with mutant-specific probes is performed with different fluorescent probs. Finally, the detection and quantification of wild-type or mutant alleles are done by flow cytometry analysis of the beads. Moreover, specific variants can be isolated by flow cytometry sorting and used for further analysis. Because BEAMing analyzes one allele at a time, it is highly sensitive for the detection of rare mutant allele which is the exact molecular environment found in ctDNA cases. It has been shown to be potential for detecting PIK3CA and EGFR mutations using ctDNA [46–48].

### 3.6. Mass Spectrometry

In combination with base extension after PCR, mass spectrometry allows ctDNA detection with single-base specificity and single DNA molecule sensitivity [49]. Briefly, DNA is first amplified by PCR and then linear amplification with base extension reaction which is designed to anneal to the region upstream of the mutation site. Few bases are added to the extension primer to produce different extension products from wild-type DNA and mutant DNA. Mass spectrometry has been applied in detection of EGFR mutations in plasma DNA from lung cancer patients [50, 51].

### 3.7. High-Resolution Melting Analysis

HRM analysis is a powerful technique for the detection of mutations, polymorphisms, and epigenetic differences using double-stranded DNA samples. Typically PCR will be used prior to HRM analysis to amplify the DNA region in which their mutation of interest lies. The HRM process is simply a precise warming of the amplicon DNA from around 50°C up to around 95°C. When the melting temperature of the amplicon is reached and the two strands of DNA separate or “melt” apart, the HRM is to monitor this melting process happening in real time. This is achieved by using fluorescent dyes that bind specifically to double-stranded DNA. When the dyes are bound, they fluoresce brightly and they only fluoresce at a low level in the absence of double-stranded DNA. The melting temperature of double-stranded DNA molecules is influenced by several factors such as the length, GC content, and sequence, which are properties of the individual molecule. Thus, the difference on DNA sequences on various mutants determines the different melting temperature and will show different HRM signatures and it was shown to be suitable for serum EGFR mutation screening for NSCLC patients [52, 53].

### 3.8. DHPLC

DHPLC uses heteroduplex formation between wild-type and mutated DNA strands to identify mutations. Heteroduplex molecules could be separated from homoduplex molecules by ion-pair, reverse-phase liquid chromatography on a special column matrix with partial heat denaturation of the DNA strands [54]. In EGFR mutation analysis, mutations in exons 18 to 21 were analyzed using a DNA endonuclease, SURVEYOR assay, which cleaved mismatched heteroduplexed DNA [55]. For these analyses DNA could be prepared from both frozen and formalin-fixed, paraffin-embedded (FFPE) tumor specimens as well as ctDNA from plasma [38, 56, 57]. Furthermore, a partially denaturing HPLC (pDHPLC) assay was developed to detect a large range of sequence variants with high sensitivity and low detection limits for minority alleles which could be a useful approach for routine detection of EGFR variants [58].

### 3.9. Next-Generation Sequencing (NGS)

Over the past years, there has been a dramatically shift away from automated Sanger sequencing to the NGS platform for genome analysis [59]. The NGS technologies include a number of methods grouped broadly as template preparation, sequencing and imaging, and data analysis. The combination of specific protocols distinguishes one platform from another that determines the data output from each platform as well as their quality and cost [60]. In addition to the pure genomic studies, the NGS technology has also been used to characterize the evolutionary relationships of ancient genomes, to elucidate the role of noncoding RNAs in disease, and to detect oncogenic mutations as well [61–64]. For the oncogenic detection application, it has been introduced into the clinical analysis and was further designed as streamlined commercial products with targeted panels which cover the main genetic alterations with predictive value, including EGFR mutations [61, 63–65]. In addition, it has been shown that NGS could also be used for ctDNA based EGFR mutation analysis [66, 67]. However, the cost is relatively higher than the PCR-based methods and the clinical usage is still limited.

## 4. Assays for EGFR Mutations Using ctDNA in Other Biofluid Samples

There are limited studies of using other noninvasive biofluid samples for detecting oncogenic mutations in lung cancer until recent studies using urine and saliva. Although somatic mutation detection in urine has previously been performed in patients with cancer, nearly all prior studies were restricted to patients with genitourinary malignancies [68–70]. Hyman and colleagues demonstrated that there was 100% concordance between tissue and urinary cfDNA genotype in treatment naïve samples from patients with systemic Histiocytic disorders using a droplet-digital PCR assay for quantitative detection of the BRAFV600E mutation [71]. Janku, one of the colleagues, further implied urinary cfDNA might have utility in detecting advanced cancer patients with BRAF-mutant tumors for treatment response [72]. They enrolled 17 patients with advanced, biopsy-proven BRAF-mutant cancers, including melanoma, nonsmall cell lung cancer, and colorectal cancer. Of these patients, 88% had the same mutation in urinary cfDNA. Longitudinal analysis further showed that changes in the amount of BRAF V600E cfDNA correlated with response to BRAF/MEK targeted therapy. Mutation detection in urine not only provides convenience for disease monitoring on an outpatient basis without the need for blood sampling but also provides flexibility of storage since DNA in urine can be stabilized for at least 9 days compared to the only 6-hour limit for accurate assessment of cfDNA in plasma [73].

Saliva contains a variety of biomolecules, including DNA, mRNA, miRNA, protein, metabolites, and microbiota. The changes in their salivary concentration can be applied to develop potential biomarkers for detecting early oral and systemic diseases including oral cancer, lung cancer, and ovary cancer as well as evaluating disease prognosis and monitoring the response to treatment [74, 75]. The salivary genome consists of both human and microbial DNAs. Nearly 70% of the salivary genome is of human origin, while the remaining 30% is from the oral microbiota [76]. The quality of salivary DNA is good that 72% to 96% of samples can be genotyped; 84% can be amplified; and 67% can be sequenced [77, 78]. In addition, it can be stored for long term without significant degradation [79]. However, no oncogene mutated DNA was identified in saliva previously. Recently, we explored the clinical utility of saliva to detect EGFR mutations in NSCLC patients by developing a core technology, electric field-induced release and measurement (EFIRM) [80]. We termed the Saliva-Based EFIRM detection of EGFR mutation as SABER. The detection of EGFR mutations by SABER was developed from cell line and validated in lung cancer xenograph model and clinical sample. And finally, a blinded test was performed on saliva from 40 late-stage NSCLC patient saliva samples. The receiver operating characteristic analysis indicated that EFIRM detected the exon 19 deletion with an area under the curve (AUC) of 0.94 and the L858R mutation with an AUC of 0.96.

## 5. Clinical Implementation of Detecting EGFR Mutations Using ctDNA in Biofluid

### 5.1. Concordance with Tissue Biopsy

When ctDNA was used to detecting EGFR mutations in NSCLC patients, one key concern was whether or not the genetic variation within ctDNA was consistent with tumor tissue. Many studies have demonstrated that blood samples could be used to reflect genetic changes in tumor of NSCLC patients (Table 1). Another key issue was what would be the best method for detecting EGFR mutations using ctDNA. For ctDNA based EGFR mutation assay, the most commonly used method is ARMS/Scorpion assay. In average, this method provided very high specificity but its sensitivity performance varied largely. Other methods such HRM and Digital PCR seemed to had better sensitivity [28, 52]. In these studies, due to different sample cohorts recruited, the results should be further verified by more comprehensive comparison studies. Concerning the comparison of the sensitivity of these methods, recently a meta-analysis study demonstrated that DHPLC and HRM showed higher sensitivity than ARMS in subgroup analyses [81]. However, in another report, DHPLC and mutant-enriched PCR showed lower sensitivity than ARMS [25]. In addition, another study also emphasized that different stage and different differentiation of cancer cell may affect the sensitivity [31]. Thus, more studies are needed to further clarify this issue.

### 5.2. Monitoring Drug Resistance

Despite good responses to EGFR TKIs in the majority of lung cancer patients carrying sensitive EGFR mutations, most of these patients eventually become resistant to EGFR TKIs within 1 year [82]. Since patients at this stage are often too weak to receive second biopsy, a noninvasive method for detecting T790M mutation remains an unmet need for directing patient treatment strategy. Though T790M mutation was identified at 2005 [83, 84], it is not until 2009 that T790M mutation was proved being identified from plasma DNA in 54% (15 of 28) of patients with prior clinical response to gefitinib/erlotinib, 29% (4 of 14) with prior stable disease, and in 0% (0 of 12) that had primary progressive disease or were untreated with gefitinib/erlotinib [19]. In other studies, activating T790M mutation was detected in 72.7% and 28% of plasma DNA using different methods [50, 85]. The progression free survival of the T790M-positive patients was proved significantly shorter than that of the T790M-negative patients [50]. However, unlike the study in using ctDNA to detect EGFR 19Del or L858R, these studies did not investigate the concordance with tumor tissue since most of these patients did not receive second biopsy.

### 5.3. Early Detection

Surgery is the most effective treatment for lung cancer but only one-third of lung cancer patients were diagnosed at early stage and amenable to surgery. Early-stage detection has the theoretical potential to reduce lung cancer mortality. Recently, National Lung Screening Trial (NLST) demonstrated that low-dose computed tomography screening (LDCT) is an effective way of detecting early lung cancer and reducing lung cancer mortality [50] compared to conventional chest X ray image. However, the study also raised two major unmet needs including the identification of nonsmoker subjects who carry the highest likelihood of developing lung cancer and which nodules are likely to be cancerous before sending patients into surgery. The NLST eligibility criteria did not clearly identify all the high-risk subjects for lung cancer who will be most likely to benefit from LDCT screening and the false positive findings confer potential harm from unnecessary interventions and undue anxiety for patients [86, 87]. Recently, using an ultrasensitive method for quantitating methods, ctDNA was detected in 100% of patients with stage II–IV NSCLC and in 50% of patients with stage I, with 96% specificity for mutant allele fractions down to ~0.02% [88]. Another study also demonstrated that EGFR DNA can be detected in early-stage lung cancer ranging from 10% to 81%. However, the application of circulating EGFR DNA for lung cancer screening should be limited on certain high risk groups. According to the International Cancer Advocacy Network (ICAN) study (NCT01106781) investigating EGFR gene mutation status in early-stage Chinese NSCLC patients with adenocarcinoma (ADC) histology, 55.1% patients were EGFR mutation positive [89]. The mutation rate is quite similar to the pioneer study, a prospective, molecular epidemiology study of EGFR mutations in Asian patients with advanced NSCLC of adenocarcinoma histology [90]. Though the detection of EGFR DNA in early lung cancer remains rare and the detection rate is lower than late stage, it remains a promising tool for screening lung cancer in combination with LDCT in Asian area.

## 6. Conclusion

ctDNA and CTC for detecting EGFR mutation have received more and more interest since a feasible, reliable, and minimally invasive approach is needed for clinical research and practice. ctDNA analysis is likely to be the preferred option for genotyping, monitoring treatment response, and early detection with no need to enrich and isolate a rare population of cells. However, optimizing and standardizing new technologies with appropriate analytical and clinical validity remained to be great challenges. In addition, other biofluids such as saliva and urine also have the potential for detecting EGFR mutations but large prospective clinical trials are needed for establishing the clinical utility. Finally, the combination of LDCT and EGFR mutation detection using ctDNA may provide an attractive method for screening early-stage lung cancer which could be the best way to decrease the high mortality of lung cancer.

## Figures and Tables

**Table 1:** Recent advancement of techniques for detecting EGFR mutation using ctDNA in lung cancer.

Study team	Sample	Oncogene mutation	Sample size	Method	Conclusion
Wang et al.	Plasma	EGFR	68 (III/IV)	ARMS/Scorpion assay	Sensitivity (22.06%), specificity (96.97%), positive predictive value (88.24%), and negative predictive value (54.70%) [20]

Liu et al.	Plasma	EGFR	86 (III/IV)	ARMS	Sensitivity (67.5%), specificity (100%), and concordance rate was 84.9% [21].

Goto et al.	Plasma	EGFR	86 (III/IV)	ARMS/Scorpion assay	Sensitivity (43.1%), specificity (100%), positive predictive value (100%), negative predictive value (54.7%), and concordance ratio (66.3%) [22]

Kimura et al.	Plasma	EGFR	42 (advanced stage)	ARMS/Scorpion assay	Sensitivity (85.7%), specificity (94.2%), and concordance ratio (92.9%) [23]

Kimura et al.	Plasma	EGFR	27 (III/IV)	ARMS/Scorpion	Detection rate 48.1% [24]

Yung et al.	Plasma	EGFR	35 (III/IV)	Digital PCR	Sensitivity (92%) and specificity (100%) [28]

Brevet et al.	Plasma	EGFR	34 (III/IV)	Mass spectrometry genotyping	Detection rate 61% [51]

Hu et al.	Plasma	EGFR	24 (I/II/III/IV)	High-resolution melting analysis	Positive rate was 100% for patients in stages II–IV, 81.8% (9/11) for stage I. The sensitivity was 91.67% and specificity was 100% [52].

Zhao et al.	Plasma	EGFR	111 (I/II/III/IV)	Mutant-enriched PCR	Concordance ratio (71.2%), sensitivity (35.6%), and specificity (95.5%). Sensitivity varied according to the disease stage and pathological differentiation; early stage (10%) versus advanced stage (56%). Highly differentiated (20%) patients and moderately differentiated (19%) and poorly differentiated subgroup (77.8%) [31].

Jiang et al.	Plasma	EGFR	58 (III/IV)	Mutant-enriched PCR	Sensitivity (77.8%), specificity (100%), and concordance rate (93.1%), more sensitive than the nonenriched assay [32].

Bai et al.	Plasma	EGFR	230 (III/IV)	DHPLC	Sensitivity 81.8% and specificity 89.5% [57]

Kim et al.	Plasma	EGFR	35 (III/IV)	PNA-mediated PCR	Concordance in the serum and tumor samples was 17% [42].

Kim et al.	Plasma	EGFR	57 (III/IV)	PNA–LNA PCR clamp	Concordance in the serum and tumor samples was 87.7% [43].

Xu et al.	Plasma	EGFR	51 (III/IV)	ARMS/Scorpion assay	Sensitivity (50.0%) Specificity (100%) [25]
				Mutant-enriched PCR	Sensitivity (25.0%) Specificity (96.2%)
				DHPLC	Sensitivity (25.0%) Specificity (92.3%)
			60 (III/IV)	Direct sequencing versus Mutant-enriched PCR	Sensitivity 18.3% versus 55.0% [33]

Kuang et al.	Plasma	EGFR −T790M	54 (III/IV)	ARMS/Scorpion assay	Detected in 54% of patients with prior clinical response to TKI and 29% of prior stable disease [19]

Taniguchi et al.	Plasma	EGFR −T790M	44 (III/IV)	BEAMing	82.6% detection rate in patient who developed PD after EGFR TKI and 43.5% detection rate in patients were never treated with EGFR TKI [48]

Sakai et al.	Plasma	EGFR −T790M	75 (III/IV)	Mass spectrometry genotyping	28% detection rate in patient who developed PD after EGFR TKI [50].

Kukita et al.	Plasma	EGFR	144 (III/IV)	Next-generation sequencers: Ion Torrent PGM	72.7% detection rate in exon 19 deletion, 78.2% detection rate in L858R or L861Q [66]

Couraud et al.	Plasma	EGFR (exons 18, 19, 20, and 21)	68 (I/II/III/IV)	Next-generation sequencers: Ion Torrent PGM	Sensitivity ranged from 55% (EGFR exon 19) to 100% (EGFR exon 18) Considering all amplicons, the sensitivity was 58% and the concordance rate was 68% [67].

Wei et al.	Saliva	EGFR	40 (III/IV)	EFIRM	Exon 19 Del (AUCs = 0.94, 95% CI, 0.82–1) and L858R (AUCs = 0.96, 95% CI, 0.90–1) [80]

ARMS: amplification-refractory mutation system; DHPLC: denaturing high performance liquid chromatography; PNA: peptide nucleic acid; PNA-LNA: peptide nucleic acid-locked nucleic acid; BEAMing: beads, emulsions, amplification, and magnetics; NGS: next-generation sequencing; Ion Torrent PGM: Ion Torrent Personal Genome Machine (PGM) System; EFIRM: electric field-induced release and measurement.
